# Defect Passivation Scheme toward High-Performance Halide Perovskite Solar Cells

**DOI:** 10.3390/polym15092010

**Published:** 2023-04-24

**Authors:** Bin Du, Kun He, Xiaoliang Zhao, Bixin Li

**Affiliations:** 1 School of Materials Science and Engineering, Xi’an Polytechnic University, Xi’an 710048, China; 2 School of Physics and Chemistry, Hunan First Normal University, Changsha 410205, China; 3 Shaanxi Institute of Flexible Electronics (SIFE), Northwestern Polytechnical University (NPU), Xi’an 710072, China

**Keywords:** perovskite solar cell, defect passivation, interface engineering, surface treatment, dopant passivation

## Abstract

Organic-inorganic halide perovskite solar cells (PSCs) have attracted much attention in recent years due to their simple manufacturing process, low cost, and high efficiency. So far, all efficient organic-inorganic halide PSCs are mainly made of polycrystalline perovskite films. There are transmission barriers and high-density defects on the surface, interface, and grain boundary of the films. Among them, the deep-level traps caused by specific charged defects are the main non-radiative recombination centers, which is the most important factor in limiting the photoelectric conversion efficiency of PSCs devices to the Shockley-Queisser (S-Q) theoretical efficiency limit. Therefore, it is imperative to select appropriate passivation materials and passivation strategies to effectively eliminate defects in perovskite films to improve their photovoltaic performance and stability. There are various passivation strategies for different components of PSCs, including interface engineering, additive engineering, antisolvent engineering, dopant engineering, etc. In this review, we summarize a large number of defect passivation work to illustrate the latest progress of different types of passivators in regulating the morphology, grain boundary, grain size, charge recombination, and defect density of states of perovskite films. In addition, we discuss the inherent defects of key materials in carrier transporting layers and the corresponding passivation strategies to further optimize PSCs components. Finally, some perspectives on the opportunities and challenges of PSCs in future development are highlighted.

## 1. Introduction

Traditional energy, which was mainly used for power generation in the past, is facing the problems of resource shortage and ecological impact. Therefore, green and renewable new energy has attracted much attention in recent years. Thereinto, the photovoltaic (PV) technology that converts light energy into electrical energy is particularly impressive due to the advantages of simple preparation of collection equipment (solar cells) and easy mass production [1,2,3]. Solar cells have been developed for three generations since the birth of Bell Labs in 1954. Although silicon-based solar cells (the first generation of solar cells) and thin film compound solar cells (the second generation of solar cells) have reached more than 23% power conversion efficiency (PCE), various factors limit their further development, such as the need for high-temperature processing, high-purity material requirements, and high manufacturing costs of photovoltaic module systems. In particular, the highly toxic raw materials required for thin film compound solar cells are not conducive to industrial mass production [4,5,6,7]. Recently developed quantum dots and dye-sensitized solar cells (third-generation solar cells) also have the disadvantages of a slow development process, a complex preparation process, and low PCE, which has led to the search for new photovoltaic materials to improve the most advanced photovoltaic technology to further develop solar cells.

Perovskite solar cells (PSCs) are the latest third-generation solar cells. After just over a decade of development, the highest PCE has soared to 25.8% [8]. The light-absorbing layer material of high-efficiency and stable PSCs is mainly composed of perovskite with ABX_3_ structure. The A-site is a monovalent organic or inorganic cation with a larger radius, including MA^+^ (methylamine ion, CH_3_NH_3_^+^), FA^+^ (formamidine ion, HC (NH_2_)_2_^+^), Cs^+^, etc., the B-site is a divalent metal cation, usually Pb^2+^, Sn^2+^, etc., and the X-site is a halogen/pseudohalogen anion, such as I^−^, Cl^−^, SCN^−^ or their mixture [9]. The A-position cation will be surrounded by BX_6_ octahedral with 12-fold coordination symmetry in the ideal perovskite structure, which makes the perovskite possess an excellent absorbance coefficient (~10^5^ cm^−1^) and long carrier diffusion length (>1 μm), which greatly reduces the processing cost and material cost of perovskite photovoltaic devices, making commercialization possible [10,11]. At the same time, by adjusting the A-site cation composition in the perovskite crystal, the perovskite has a band gap adjustability (1.2–3.0 eV) beyond the reach of other photovoltaic devices, which also makes the Shockley-Queisser (S-Q) theoretical efficiency limit of perovskite/silicon tandem solar cells and perovskite/perovskite tandem solar cells exceed 30%, far more than that of single-junction solar cells [12,13]. In 2009, a pioneering work by Miyasaka et al. reported a 3.8% PCE PSCs based on liquid electrolyte using the halide perovskite material MAPbI_3_ [14]. In 2012, Park et al. first used a solid-state semiconductor 2,2′,7,7′-tetrakis[*N*,*N*-di(4-methoxyphenyl)amine]-9,9′-spirobifluorene (spiro-OMeTAD) as a hole transport layer material to successfully prepare the first all-solid-state PSCs with a PCE of 9.7% and no encapsulation [15]. Since then, PSCs based on organic-inorganic halide perovskite absorbing layers have been getting more and more attention from researchers, and the certified PCE is increasing year by year [16,17,18]. In 2021, Seok et al. obtained a PCE of 25.5% by interfacial modulation, which was a world record that year [19]. In 2022, You et al. eventually obtained a 25.6% champion-certified PCE by introducing the additive RbCl to stabilize the perovskite phase [20].

Planar PSCs devices typically consist of transparent conductive oxide (TCO) electrodes (indium tin oxide (ITO) or fluorine-doped tin oxide (FTO)), electron transport layer (ETL), perovskite light-absorbing layer, hole transport layer (HTL), and metal electrodes (Au, Ag, Cu). Planar PSCs can be divided into n-i-p planar structures and p-i-n planar structures according to the different locations of carrier transport layer distribution, where TCO electrodes/ETL/perovskite absorbing layer/HTL/metal electrodes device structure is n-i-p planar structures, TCO electrodes/HTL/perovskite absorbing layer/ETL/metal electrodes device structure is p-i-n planar structures. Although these two PSCs have different structures, they both convert light energy into electrical energy by using the photovoltaic effect of the semiconductor PN junction. In PSCs, the perovskite light-absorbing layer (intrinsic semiconductor) forms a PN junction with adjacent ETL (n-type semiconductor) and HTL (p-type semiconductor). The PN junction can absorb a large number of photons to generate excitons under the irradiation of sunlight, and the perovskite with high light absorption coefficient can absorb a large number of photons to generate excitons. The exciton binding energy is low, and it is easy to dissociate into electrons and holes. These carriers (electrons and holes) will move under the action of the PN junction and reach the two ends of the electrode through different transport layers to form a photovoltage. Once the electrode is connected, it will form a current loop to convert light energy into electrical energy [21].

**Figure 1:**
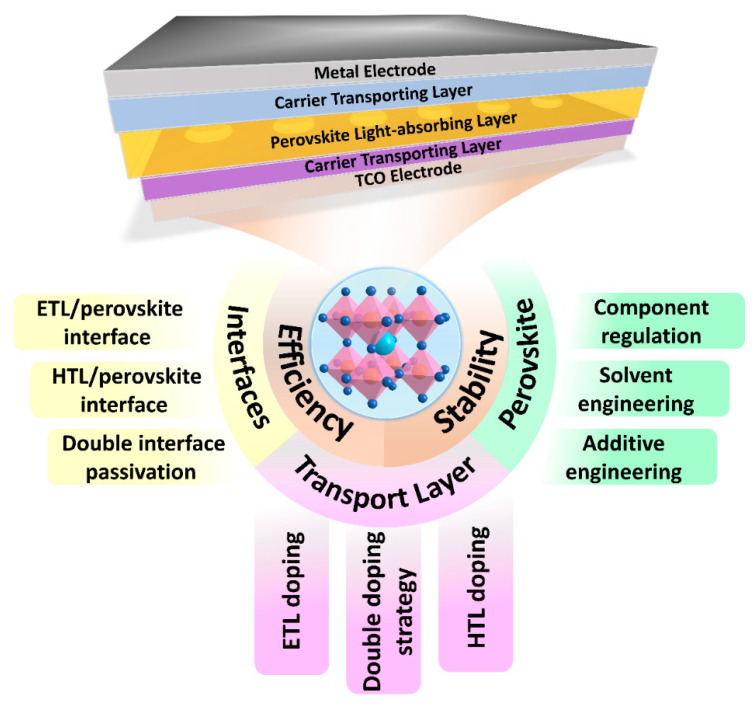
Planar PSCs device structures and passivation strategies based on different components of PSCs to improve device efficiency and stability.

The ideal crystal structure is in a state where each atom is in its corresponding position, but the actual crystal structure will be more or less affected by the defects introduced during crystal growth and the post-treatment process. The spin-coating preparation process and post-annealing process of PSCs devices will form various defects on the surface or grain boundaries of polycrystalline perovskite crystals, including (1) insufficiently coordinated anions or cations; (2) inherent point defects caused by the preparation process, such as halide vacancies, cation vacancies, and Pb-I antisites; (3) ion migration at grain boundaries; and (4) exogenous impurities [22]. These defects with positive or negative charges will introduce transition energy levels in the band gap. When the transition energy level is located in one-third of the band gap, deep-level defects will be formed, which is the source of the notorious trap-assisted recombination (also known as Shockley–Reid–Hall recombination; SRH). SRH recombination is not conducive to the extraction and migration of carriers in perovskite films and affects the lifetime of carriers, which has been identified as an important reason for limiting open circuit voltage (V_OC_), short circuit current density (J_SC_), and fill factor (FF) in PSCs [23,24]. In addition, there are more defects in the carrier transport interfaces, so it is necessary to passivate the defects of each component of the PSCs to improve the efficiency and stability of the device (Figure 1).

This review focuses on the progress and challenges of a series of passivation strategies that can effectively eliminate the main defects in PSC devices. Based on the passivation strategies for different components of PSCs, we highlight the latest research on interface engineering, perovskite surface treatment, and dopant passivation, including ETL (HTL)/perovskite interface passivation, the solvent component, precursor additive engineering, anti-solvent engineering, and ETL (HTL) doping. We summarized the important role of different functional groups in the defect passivation process. Finally, some perspectives on the opportunities and challenges of PSCs in future development are put forward.

## 2. Interface Engineering

It can be seen from the working principle of PSCs that the generation, transmission, and extraction of free carriers will go through multiple interfaces, including TCO electrode/ETL interface, ETL/perovskite interface, perovskite/HTL interface, and HTL/metal electrode interface. However, there will be a severe interface recombination process at the ETL (HTL)/perovskite interface. On the one hand, the recombination is caused by the ineffective matching of energy levels between interfaces. On the other hand, the interface defects from the carrier transport layer and the perovskite layer itself are also the main recombination centers [25,26]. The interface engineering can simply and effectively adjust the interface energy level mismatch to overcome the interface loss and also optimize the interface morphology for carrier transport [19]. In view of this, the passivation strategy of improving V_OC_ and FF by using interface engineering to minimize interface recombination has been widely studied in recent years.

### 2.1. ETL/Perovskite Interface Passivation

**Figure 2:**
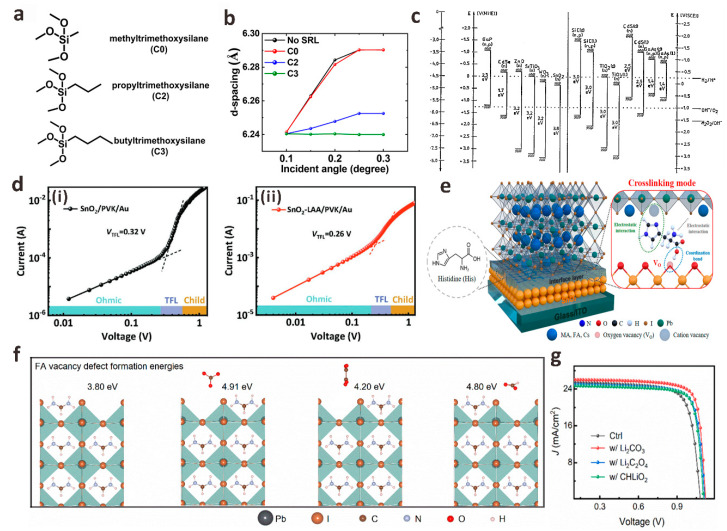
(**a**) Chemical structure of methoxysilane compound used as SRL. (**b**) GIWAXS was used to measure the (110) interplanar spacing of CsPbI_3_ deposited on different SRLs at a critical angle of 0.24°. Reproduced with permission [29]. Copyright 2022, American Chemical Society. (**c**) The band position of the commonly used electron transport layer. Reproduced with permission [31]. Copyright 1996, American Chemical Society. (**d**) Dark I-V curves of devices with different structures: (**i**) FTO/SnO_2_/PVK/Au, (**ii**) FTO/SnO_2_-LAA/PVK/Au. Reproduced with permission [32]. Copyright 2022, Wiley-VCH. (**e**) A schematic diagram of the formation of His molecules between the SnO_2_ layer and the perovskite layer. Reproduced with permission [33]. Copyright 2022, American Chemical Society. (**f**) Calculation of the energy (from left to right) of formation of FA vacancy defects before and after adsorption of CO_3_^2−^, C_2_O_4_^2−^ and HCOO^−^ ions on the FAPbI_3_ (001) surface. (**g**) J-V curves of PSCs with and without lithium salt modification. Reproduced with permission [34]. Copyright 2022, American Chemical Society.

ETL in PSCs is generally an n-type semiconductor material, which is used to extract the electrons formed by dissociation in the perovskite layer and transport them to the TCO electrode (n-i-p planar structures) or the metal electrode (p-i-n planar structures) while blocking the holes in the perovskite layer to avoid carrier recombination [27]. It has been reported that the ETL/perovskite interface contains a large number of deep defects limiting the efficiency and stability of PSCs devices, which is about 100 times greater than that of the perovskite layer defects [28]. Meanwhile, the energy level matching between ETL and perovskite layer is crucial to improve the carrier extraction and collection efficiency and the V_OC_ of the device. TiO_2_ is the earliest ETL material used in various n-i-p planar structures PSCs. Loo et al. found that the *β*-CsPbI_3_ lattice near the TiO_2_/perovskite interface will undergo polymorphic transformation under illumination, which is due to the strain at the TiO_2_/CsPbI_3_ interface [29]. In order to suppress this lattice distortion, three alkyltrimethoxysilane derivatives (C0 = methyltrimethoxysilane, C2 = propyltrimethoxysilane, and C3 = butyltrimethoxysilane) with different alkyl chain lengths were inserted between the TiO_2_/CsPbI_3_ interface as strain release layers (SRL). The results of depth-resolved grazing-incidence wide-angle X-ray scattering (GIWAXS) show that the C3 with the longest alkyl chain provides a more flexible interface, which can effectively reduce the thermal expansion mismatch between the TiO_2_/CsPbI_3_ interface, reduce the interface stress and improve the phase stability and device stability of the interface (Figure 2a,b). Compared with the original TiO_2_/CsPbI_3_ device, the PCE of TiO_2_/C3/CsPbI_3_ device increased from 15.7% to 20.1%. Wu et al. introduced cystamine dihydrochloride (CMDR) with double amino groups between dense TiO_2_ and perovskite layer to modify the interface [30]. The diamino group of CMDR can not only form Ti-N bond with TiO_2_ but also form hydrogen bond with I^−^ in perovskite, which effectively inhibits the generation of excessive metal reduction of lead (Pb^0^) defects in PbI_2_. This bilateral synergistic passivation strategy effectively reduces the contact resistance, trap state density and non-radiative recombination of carriers between the interfaces of perovskite films and finally increases the PCE from 18.41% to 20.63%.

Compared with dense TiO_2_, SnO_2_ has higher electron mobility, wider band gap, lower photocatalytic activity and preparation temperature, and, most importantly, better matching with the energy level of perovskite. Therefore SnO_2_ is one of the most ideal ETL materials for planar PSCs (Figure 2c) [31,35]. Nevertheless, the process of preparing the device will inevitably lead to the film having obvious pinholes or traps, which will lead to serious carrier recombination at the interface, resulting in a sharp decrease in device performance, so the post-processing of the SnO_2_ layer is particularly important [36]. To solve the problem that oxygen vacancies (V_O_) and hydroxyl defects on SnO_2_ ETL damage perovskite films during the preparation of PSCs, Zhang et al. used multifunctional amino acid L-aspartic acid (LAA) to “match” the SnO_2_/perovskite interface [32]. The -COOH in LAA can coordinate the mismatched Sn^4+^ in SnO_2_, thereby reducing the V_O_ defect of SnO_2_ and also neutralizing the alkalinity of the hydroxyl group on the side of SnO_2_. The amino group of LAA itself connects the perovskite layer through hydrogen bonds to improve the quality of the perovskite film. LAA forms a “channel” between SnO_2_/perovskite through bilateral synergistic passivation, accelerates electron transfer at the interface, and reduces the density of trap states at the interface (Figure 2d). When the V_OC_ is 1.15 V, the PCE of SnO_2_/LAA-based PSCs is up to 22.73%, which is much higher than 20.02% of SnO_2_-based PSCs. Similarly, Tao et al. also attempted to use the designed multifunctional histidine (His) as a cross-linking agent for the SnO_2_/perovskite interface [33]. This close cross-linking of SnO_2_ with perovskite facilitates the extraction and transfer of electrons, improves the quality of perovskite films, and reduces non-radiative recombination between interfaces (Figure 2e). Crosslinking agents can also effectively adjust the interface level and accelerate electron transfer. Finally, the PSC device based on His-modified SnO_2_ produced a champion PCE of 22.91%, V_OC_ of 1.17 V, J_SC_ of 24.21 mA cm^−2^, and FF of 80.9%.

**Figure 3:**
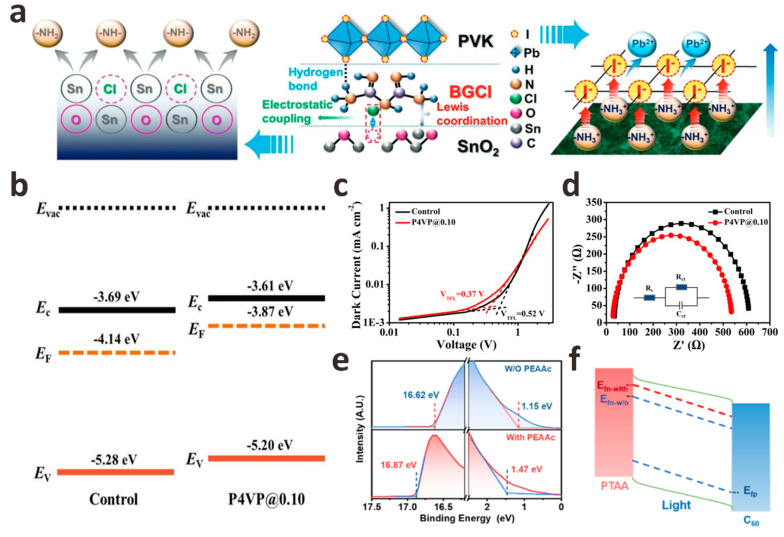
(**a**) A schematic diagram of the passivation mechanism of BGCl at the ETL/perovskite interface. Reproduced with permission [38]. Copyright 2022, Wiley-VCH. (**b**) Control and P4VP modified perovskite film energy level diagram. (**c**) Dark J-V curve of a pure electronic device with a device structure of ITO/SnO_2_/MAPbI_3_/P4VP/PCBM/Al. (**d**) In dark conditions, the EIS of the controlled and P4VP-modified device is measured at 640 mV, and the illustration shows the equivalent circuit for fitting the impedance spectrum data. Reproduced with permission [39]. Copyright 2020, The Royal Society of Chemistry. (**e**) UPS spectra of perovskite films with and without PEAAc treatment. (**f**) Schematic diagram of Fermi level splitting under illumination. Reproduced with permission [40]. Copyright 2022, American Chemical Society.

In addition to these monomolecular layers that act as interface crosslinking agents, salt molecules containing both anions and cations show great potential in regulating the ETL/perovskite interface energy level, and if necessary, the ETL/perovskite interface layer is modified by customizing salt molecules of anions (cations) with specific passivation functions. By studying the effects of a series of lithium salt anions (CO_3_^2−^, C_2_O_4_^2−^, and HCOO^−^) on the SnO_2_ layer, FAPbI_3_ layer, and SnO_2_/FAPbI_3_ interface, Bi et al. developed a strategy for Li salt molecules to passivate the SnO_2_/FAPbI_3_ interface [34]. They found that when the C-O group and C=O group of these anions are in optimal configurations, they will coordinate with the uncoordinated Sn^4+^ and FA^+^ on both sides of the interface to form stronger bonds. The results of density functional theory (DFT) calculations (Figure 2f) show that compared with C_2_O_4_^2−^ and HCOO^−^, the binding energy between CO_3_^2−^ and FA^+^ is the strongest, which increases the formation energy of V_FA_ defects and releases the residual stress of FAPbI_3_ lattice. The modification of Li_2_CO_3_ greatly promoted the charge transfer between the SnO_2_/FAPbI_3_ interface, effectively reduced the carrier recombination between the interfaces, optimized the crystallinity of the perovskite film, and finally obtained FAPbI_3_ PSCs with a PCE of 23.5% (Figure 2g). In addition to Li^+^, guanidine cations (GA^+^) are also widely used in SnO_2_/FAPbI_3_ interface defect passivation to prepare high-quality perovskite films. Zang et al. prepared different guanidinium salts (GASCN, GASO_4_, GAAc, and GACl) by using a series of anions (SCN^−^, SO_4_^2−^, Ac^−^ and Cl^−^) that have a positive effect on the surface defect passivation, interface energy level matching, and the crystallization of PbI_2_ and perovskite layers [37]. The effects of different anions on the interfacial chemical interaction strength, trap density, film crystallinity, and energy level on the device performance were systematically studied. The results show that all interface passivators can effectively passivate the defects on the surface of SnO_2_ and perovskite, adjust the interface band alignment, and promote the crystallization of perovskite. The passivation effect of anions from SCN^−^, SO_4_^2−^, and Ac^−^ to Cl^−^ is getting better. Compared with 21.84% of the control device, the device champion PCE modified by GASCN, GA_2_SO_4_, GAAc, and GACl was 22.76%, 23.43%, 23.57%, and 23.74%, respectively. In addition, the thermal stability and environmental stability of the modified equipment were also improved. Sun et al. used biguanide hydrochloride (BGCl) as a multifunctional interfacial modifier to simultaneously optimize charge extraction and transport at the SnO_2_/perovskite interface and promote the growth of perovskite crystals, confirming the synergistic passivation effect of BGCl (Figure 3a) [38]. Firstly, The N in BGCl is coordinated with the uncoordinated Sn^4+^ in SnO_2_ by Lewis coupling in an alkaline environment. With the injection of electrons on the surface of SnO_2_, the electron extraction and transport capacity at the SnO_2_/perovskite interface is significantly enhanced. In addition, Cl^−^ ions in BGCl occupy the oxygen vacancy (V_O_) through electrostatic coupling, thereby reducing the V_O_ density on SnO_2_. Secondly, -NH_2_/-NH_3_^+^ in BGCl can be anchored to I/I^−^ in PbI_2_ by hydrogen bonding to achieve uniform perovskite crystal growth. After BGCl modification, the interface defects of SnO_2_/perovskite are effectively passivated, and the quality of perovskite film is improved. Finally, 24.4% certified PCE is achieved. This work provides an effective method for the selection and design of interface modification molecules.

The interface recombination at the ETL/perovskite interface is also important in p-i-n PSCs. Liu et al. introduced poly-4-vinylpyridine (P4VP) as an intermediate film between the perovskite/[6,6]-phenyl-C61-butyric acid methyl ester (PCBM) interface to passivate defects existing on the surface and grain boundaries [39]. The results show that P4VP effectively adjusts the energy level matching of perovskite/PCBM, which is conducive to efficient charge extraction between interfaces and inhibits hole transfer. At the same time, the space-charge-limited current (SCLC) and electrochemical impedance spectroscopy (EIS) characterization results further confirmed that P4VP could effectively passivate surface defects (Figure 3b–d). Finally, the PCE of PSCs modified by P4VP increased from 17.46% to 20.02%. Based on the introduction of the ethanediamine dihydroiodide (EDAI_2_) interface passivation layer, Ding et al. introduced the hexamethylene diisocyanate (HDI) interface layer to further treat the perovskite/PCBM interface [41]. Their results show that the interface recombination after EDAI_2_/HDI passivation is significantly inhibited, and a very low non-radiative V_OC_ loss of 0.10 V is obtained. They verified that the isocyanate group in the HDI molecule could easily cross-link with the amine group in EDAI_2_ even at room temperature, and the cross-linking molecule is formed on the surface of the perovskite, which helps to hinder the diffusion of EDA^2+^ cations into the perovskite, making EDAI_2_/HDI passivated PSCs have excellent thermal stability. Xu et al. introduced an ultra-thin interface layer of phenylethylammonium acetate (PEAAc) at the wide band gap perovskite/C60 interface, which effectively alleviated the surface defects of perovskite coordination and greatly reduced the non-radiative recombination loss [40]. Through ultraviolet photoelectron spectroscopy (UPS), they found that the Fermi level of perovskite moved upward after PEAAc treatment, indicating that there were more n-type perovskite crystal planes on the surface of perovskite, which was conducive to electron extraction and hole blocking at the perovskite/C60 interface. More importantly, the higher Fermi level of the PEAAc-treated perovskite film can lead to a larger splitting between the electron quasi-Fermi level and the hole quasi-Fermi level under illumination, which contributes to V_OC_ enhancement (Figure 3e,f). Therefore, the PEAAc-modified wide-bandgap (1.68 eV) PSCs device achieved a champion PCE of 20.66% with a high V_OC_ of 1.25 V, which is one of the highest V_OC_ values reported in wide-bandgap perovskite devices in recent years.

### 2.2. HTL/Perovskite Interface Passivation 

**Figure 4:**
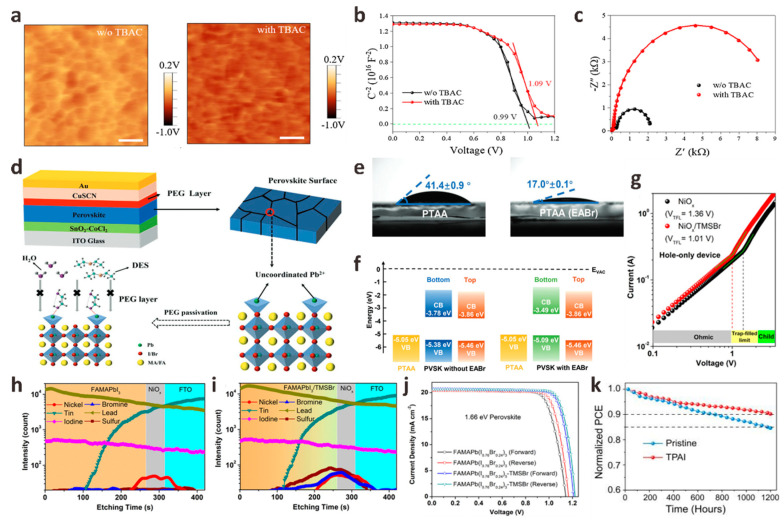
(**a**) Control (left) and TBAC-modified (right) KPFM images of perovskite films. (**b**) C-V characterization of the Mott-Schottky diagram and (**c**) EIS spectrum Nyquist diagram of the control device and the TBAC-PSCs device. Reproduced with permission [46]. Copyright 2022, Elsevier. (**d**) The schematic diagram of CuSCN-based PSCs device, the chemical structure of PEG and the coordination diagram of PEG and perovskite. Reproduced with permission [50]. Copyright 2022, Wiley-VCH. (**e**) Contact angles of perovskite precursor solution droplets on original PTAA (left) and EABr modified PTAA (right). (**f**) Energy level diagrams of PTAA and two perovskite films. Reproduced with permission [51]. Copyright 2022, Wiley-VCH. (**g**) Dark C-V curves of pure hole devices with and without TMSBr treatment, from left to right, are the low bias voltage region (Ohmic region), trap filling state region, and SCLC state region. ToF-SIMS curves of FAMAPbI_3_ perovskite films on different substrates: (**h**) FTO/NiO*_x_* and (**i**) FTO/NiO*_x_*/TMSBr. (**j**) J-V curves of FAMAPb(I_0.76_Br_0.24_)_3_ PSCs before and after TMSBr modification. Reproduced with permission [52]. Copyright 2022, The Royal Society of Chemistry. (**k**) The operating stability curves of the original and TPAI-modified unpackaged devices under an ambient atmosphere. Reproduced with permission [53]. Copyright 2022, The Royal Society of Chemistry.

HTL is usually a p-type semiconductor material. Except for blocking electrons and improving hole mobility, the most important role of HTL is to improve the stability of PSC devices. It has been reported that the presence of HTL in PSCs can increase stability by 90% [42]. The HTL/perovskite interface passivation strategy is also as important as ETL/perovskite interface passivation. For n-i-p planar structures PSCs, the first thing to consider is that the interface passivation material is directly deposited on the perovskite film, so the solvent for dissolving the interface passivation material must be an inert solvent that cannot destroy the perovskite film. The appropriate interface passivator must have the ability to passivate the surface defects of the perovskite film, increase the hole transfer rate and match the energy level between the HTL and the perovskite layer [43,44,45]. Halide anions or pseudohalide anions have been shown to react chemically with anion vacancies or cation defects on the surface of perovskite films through ionic bonds or hydrogen bonds, thereby increasing the crystallinity of perovskite films. Kong et al. introduced a tetrabutylammonium chloride (TBAC) monolayer at the perovskite/spiro-OMeTAD interface by a simple solution method [46]. When TBAC is deposited on the perovskite film, the Cl^−^ in TBAC will enter the perovskite lattice by occupying the I^−^ vacancy in the film or acting as a gap, which makes TBAC have a strong interface dipole to promote the built-in electric field and reduces the contact barrier for hole extraction. They studied the self-assembly behavior of the TBAC interface layer on the perovskite surface by Kelvin probe force microscopy (KPFM) and capacitance-voltage (C-V) tests and found that the built-in electric field induced by the TBAC dipole layer was significantly enhanced, which was further confirmed by EIS results (Figure 4a–c). Finally, the n-i-p PSCs based on the ITO/SnO2/perovskite/TBAC/spiro-OMeTAD/Au structure achieved a 23.5% champion PCE. Song et al. used phenethylammonium fluoride (PEAF) deposited on perovskite films by rapid thermal evaporation as an interfacial passivator for PSCs [47]. Fluoride anions in PEAF have a small ionic radius and high Lewis basicity, which can form strong hydrogen bonds with organic cations in perovskite N-H---F and uncoordinated Pb^2+^ to form strong ionic bonds. The quality of the perovskite film modified by PEAF is significantly improved. At the same time, the lifetime of the treated perovskite film is significantly longer (153 ns) than that of the original perovskite film (20 ns), which indicates that PEAF effectively passivates perovskite defects and reduces non-radiative recombination. Finally, the PSCs treated with PEAF had a high PCE of 23.2%, and the stability was significantly enhanced. Inspired by this work, Pan et al. innovatively synthesized hexadecyltrimethylammonium hexafluorophosphate (HTAP) by using pseudo-halide anion PF6− and hexadecyltrimethylammonium cation (HTA^+^) and coated it on the top of perovskite layer to achieve a terminal sealing strategy [48]. This strategy can provide a good “channel” for hole extraction and provide a defect passivation layer for enhancing V_OC_ and FF. PF_6_^−^ can fill the halide anion vacancies on the perovskite film and anchor the uncoordinated Pb^2+^, helping to improve the crystallization and morphology of the perovskite film. In addition, HTA with an ultra-long chain can prevent the erosion of water molecules and effectively enhance the resistance of the device to environmental erosion. They showed the macroscopic color changes of the devices after modification under light immersion. The results revealed that the control devices were seriously discolored and decomposed while the modified devices did not change significantly, indicating that HTAP sealing can effectively improve the optical stability of PSCs. The optimal device modified by HTAP obtained 23.14% of the champion PCE, and the lead leakage was effectively alleviated. Similar to PF_6_^−^, Li et al. studied the role of pseudohalide anion SCN^−^ in the passivation of perovskite/HTL interface to obtain high-quality perovskite films. They synthesized a new bifunctional material acetamidine thiocyanate (AASCN), which showed the synergistic passivation effect of polar cations and pseudohalide anions [49]. After AASCN enters the perovskite film, the polar AA^+^ containing four rotation-restricted C-N bonds can improve the stability of the perovskite, and the N-H bond of AA^+^ can effectively passivate the film by forming hydrogen bonds N-H---I with iodine vacancies in the perovskite. SCN^−^, with a small size and high structural freedom, can interact with Pb-I octahedron, and then through the Ostwald ripening process, the crystallization of perovskite films can be improved during the secondary crystal growth process to obtain higher quality perovskite films. Therefore, the PCE of FA_0.25_MA_0.75_PbI_3_ PSCs increased from 21.43% to 23.17%, and the V_OC_ increased from 1.095 V to 1.167 V.

In addition to the most common spiro-OMeTAD, inorganic p-type semiconductor cuprous thiocyanate (CuSCN) has attracted much attention as a new type of HTL. It has good transparency in the entire visible and infrared regions, good chemical stability, and higher hole mobility than spiro-OMeTAD [54]. However, devices based on CuSCN HTL have suffered from serious interface recombination problems. In order to solve the problem of interface instability, Long et al. added polyethylene glycol (PEG) as an intermediate film at the perovskite/CuSCN interface, effectively avoiding direct contact between perovskite and CuSCN to prevent SCN^−^ from destroying the perovskite crystal structure [50]. PEG can anchor MA^+^ and I^−^ in perovskite to inhibit ion migration, improve the poor contact between the perovskite and CuSCN interface, enhance the hole mobility of the perovskite film, and passivate the uncoordinated Pb^2+^ in the perovskite film to inhibit the Pb^0^ defects (Figure 4d). At the same time, the unique hygroscopicity of PEG molecules can form a water barrier around the perovskite film, effectively enhancing the environmental stability of PSCs. Through this new interface engineering, an excellent PCE of 19.20% was finally achieved. This is one of the most efficient standards reported to date in CuSCN-based PSCs. This work broadens the prospects for the commercialization of efficient and stable CuSCN-based PSCs.

The HTL/perovskite interface passivation of p-i-n planar structures PSCs has been rarely studied in the past. However, similar to the HTL/perovskite interface passivation in the n-i-p structure, the HTL/perovskite interface passivation in the p-i-n structure will also greatly affect the final device performance. Poly(bis{4-phenyl}{2,4,6-trimethylphenyl}amine) (PTAA) is one of the most commonly used HTL semiconductors for p-i-n PSCs. However, the large surface tension and incomplete surface coverage between PTAA and perovskite films are severe challenges for the preparation of high-performance PSCs. In this regard, Wu et al. introduced an inorganic potassium fluoride (KF) interfacial buffer layer onto the PTAA substrate to adjust the surface energy level difference between PTAA and perovskite [55]. KF can effectively reduce the valence band maximum (VBM) of PTAA, which is beneficial to the extraction of holes. In addition, the introduction of the KF layer significantly increases the composite resistance of the PTAA/perovskite interface, thereby inhibiting the carrier recombination between the interfaces. Finally, the PSCs modified by KF showed a PCE of 21.51%, J_SC_ of 23.95 mA cm^−2^, V_OC_ of 1.09 V, and FF of 82.4%. Xing et al. introduced ethylammonium bromide (EABr) into the bottom interface of perovskite films to study its passivation effect [51]. The water contacts angle of the EABr-modified perovskite film decreased from the original 42° to 17°, indicating that the wettability of the EABr-modified PTAA substrate was improved, which was beneficial to the growth of the perovskite film. In addition, the ammonium group in EABr can significantly reduce the unreacted PbI_2_ crystals at the PTAA/perovskite interface. These crystals have been reported to be the main defect sources and main degradation sites of perovskite films. The introduction of EABr also moves the VBM at the bottom of the perovskite upward by 0.29 eV, which improves the energy level alignment between perovskite and PTAA and promotes the extraction of holes (Figure 4e,f). Finally, the PSCs modified based on the EABr interface layer achieved a V_OC_ of 1.20 V, the champion PCE also increased from 20.41% to 21.06%, and the stability was also improved. Another work of Xing et al. is the introduction of donor-acceptor-donor organic molecule 4,4′,4′′-(1-hexyl-1*H*-dithieno [3′,2′:3,4; 2′′,3′′:5,6] benzo [1,2-d] imidazole-2,5,8-triyl) tris (*N*,*N*-bis(4-methoxyphenyl) aniline (M2) to try to alleviate the inherent hydrophobicity of PTAA, which hinders the production of high-quality perovskite films on PTAA substrates [56]. The wettability of PTAA after M2 modification is greatly improved, and the crystallinity of the perovskite film formed on this substrate is significantly enhanced. More importantly, due to the excellent hole extraction and transport properties of M2, PTAA/M2 also exhibits higher hole mobility and conductivity than the original PTAA. The introduction of the M2 layer can also reduce the highest occupied molecular orbital (HOMO) energy level gap between PTAA and perovskite, thereby reducing the Voc loss. Therefore, the champion PCE of p-i-n PSCs based on PTAA/M2 increased from 18.67% to 20.23%, and the operational stability and light stability were enhanced.

Due to its high carrier mobility and high transmittance, nickel oxide (NiO*_x_*) has become a common HTL in p-i-n PSCs in addition to PTAA. However, recent reports have pointed out that the photo-induced degradation of NiO*_x_*-perovskite heterojunction is the main factor limiting the life of NiO*_x_*-based PSCs devices [57]. For this reason, Qi et al. used vapor deposition to introduce a trimethylsulfonium bromide (TMSBr) buffer layer between the NiO*_x_*/perovskite interface to eliminate the multi-step photodegradation of the NiO*_x_*-perovskite heterojunction accompanying the device preparation process [52]. Time-of-flight secondary ion mass spectrometry (ToF-SIMS) results confirmed the penetration of TMS^+^ and Br into the perovskite layer. The TMSBr buffer layer can eliminate the deprotonation and redox reaction between the organic iodide in the perovskite precursor and the Ni^3+^ in the NiO*_x_* layer, which greatly improves device efficiency and stability. At the same time, the TMSBr buffer layer also has lattice parameters matching with perovskite crystals and strong trap passivation ability. TMS^+^ in TMSBr can also significantly delay the proton transfer process at the NiO*_x_*/perovskite interface. Finally, the p-i-n PSCs with TMSBr buffer layer achieved a champion PCE of 22.1%, and the time to reduce the efficiency to 80% of its initial value under AM 1.5G illumination was 2310 h, which is one of the highest service life reported by NiO*_x_*-based PSCs (Figure 4g–j). In addition, the mismatch of the thermal expansion coefficient leads to the residual strain caused by the interface between NiO*_x_* and perovskite, which accelerates the degradation of perovskite film and reduces the stability of the device. In this regard, Yang et al. introduced a tetrapentylammonium iodide (TPAI) buffer interface by sequential deposition method to prepare strain-free hybrid perovskite film [53]. Due to the low interaction energy between its flexibility and perovskite, the TPAI buffer layer can effectively release the in-plane tensile stress of PbI_2_, thereby expanding the PbI_2_ layer spacing, which is beneficial to release the residual stress between NiO*_x_* and PbI_2_ films in the subsequent perovskite phase transformation and accelerate the transformation of PbI_2_ to perovskite. The TPAI buffer layer can also passivate interface defects, increase hole mobility, and improve device stability (Figure 4k). The champion PCE of IPAI-modified MAPbI_3_ PSCs is 22.14%, with an FF of 84.6%. This work paves a new way to fabricate strain-free hybrid PSCs.

## 3. Perovskite Surface Treatment

As the light-absorbing layer, perovskite thin films are the most important part of PSCs devices. Perovskite thin films with high phase purity, low structural defects, excellent morphology, and high crystallinity are the key factors in obtaining high-efficiency PSCs. However, the perovskite crystal structure will be more or less affected by crystal growth and the post-treatment process, resulting in defects in the crystal [58,59,60]. To achieve high PCE and stability, it is very important to further improve the quality of perovskite films. It is urgent to eliminate SRH recombination in perovskite films. Generally, the most effective passivation methods are the solvent component, precursor additive engineering, and anti-solvent engineering methods.

### 3.1. The Solvent Component

With the rapid development of PSCs, the spin-coating process has become the most effective film manufacturing method, and various modified deposition based on the spin-coating process has achieved good performance [61,62]. Perovskite precursor solution plays an important role in the crystallinity, morphology, and stoichiometry of perovskite films. The solvent in the precursor solution can control perovskite nucleation and crystal growth to achieve uniform, pinhole-free high-quality perovskite films [63]. Precursor solutions contain inorganic and organic precursors with different properties, allowing only a limited selection of common solvents with sufficient solubility for the vast majority of mixtures [64]. Since the advent of PSCs, finding solvents or solvent mixtures with the appropriate properties to significantly increase the performance and stability of PSCs devices has been a major goal for many researchers.

In the early research stage of PSCs, the preparation of perovskite films mainly focused on the use of a single solvent perovskite precursor solution. At this time, a unipolar solvent such as *N*,*N*-dimethylformamid (DMF) or γ-butyrolactone (GBL) is typically used to prepare a perovskite precursor solution. The solution is spin-coated onto the substrate to form a wet perovskite precursor film, which is then converted into a perovskite film by annealing to remove the solvent [65,66]. Since the solubility of PbI_2_ in DMF or GBL is relatively poor, and the weak coordination between PbI_2_ and DMF or GBL makes it very easy for PbI_2_ to crystallize preferentially from the precursor solution during the spin coating process, the morphology of the prepared perovskite film is always poor. To overcome this problem, Han et al. prepared an MAPbI_3_ precursor solution by using dimethyl sulfoxide (DMSO) as an alternative precursor solvent [67]. The results show that DMSO can effectively delay the rapid crystallization of PbI_2_, thus overcoming the problem of incomplete conversion of PbI_2_. A high PCE planar PSC of 13.5% was prepared. Nevertheless, DMSO without high viscosity cannot be used as a single precursor solvent in one-step deposition methods. Further studies confirm that the use of a single solvent for perovskite film deposition is not the best choice for precise control to achieve high-quality films with the disadvantage of low nucleation rate and fast crystal growth, resulting in the formation of needle-like crystals in the film. The incomplete coverage of the film in the deposition area not only reduces the active area of the light-absorbing layer and induces direct contact between the interfaces but also leads to the intensification of perovskite SRH recombination. To overcome this shortcoming, Seok et al. first proposed the use of a mixed solvent of GBL and DMSO to prepare a perovskite precursor solution [68]. Through the PbI_2_/MAI@DMSO mesophase, a very uniform and dense perovskite film is formed, and PSCs with a champion PCE of 16.2% can be fabricated. Subsequently, a series of mixed solvents, including DMF, DMSO, *N*-methyl-2-pyrrolidone (NMP), and GBL, were used as perovskite precursor solvents. So far, DMF/DMSO mixed solvent strategy has been widely used in the precursor solution of high-efficiency PSCs [69,70].

**Figure 5:**
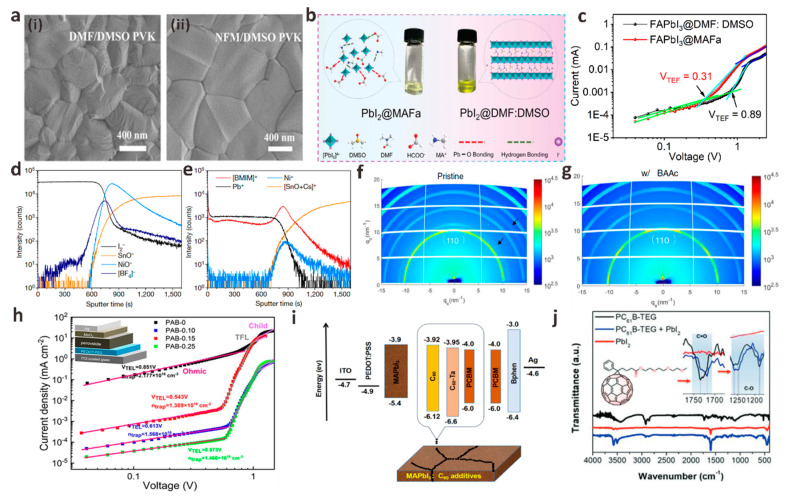
(**a**) SEM images of perovskite films with different precursor solvents: (**i**) DMF/DMSO and (**ii**) NFM/DMSO. Reproduced with permission [73]. Copyright 2022, American Chemical Society. (**b**) PbI_2_@MAFa and PbI_2_@DMF/DMSO solution image and solution interaction diagram. (**c**) The J-V characteristic curves of pure electronic devices based on FAPbI_3_@DMF/DMSO perovskite film (prepared in an N_2_-filled glove box) and FAPbI_3_@MAFa perovskite film (prepared at 70–90% humidity). Reproduced with permission [74]. Copyright 2021, American Association for the Advancement of Science. The ToF-SIMS depth profiles of perovskite films on FTO/NiO/BMIMBF_4_ substrates are measured with (**d**) negative and (**e**) positive polarity. Reproduced with permission [75]. Copyright 2019, Springer Nature. (**f**) Control and (**g**) 2D GIWAXS images of BAAc-treated perovskite films, where the diffraction rings at about 11 nm^−1^ and 16 nm^−1^ are marked with black arrows. Reproduced with permission [76]. Copyright 2022, Wiley-VCH. (**h**) Dark J-V curves of pure hole devices with different contents of PAB-modified perovskite layers [77]. Copyright 2022, Elsevier. (**i**) Energy level diagram of PSCs modified by different fullerene derivative materials. Reproduced with permission [78]. Copyright 2020, Elsevier. (**j**) FTIR spectra of PbI_2_ interacting with PC_61_B-TEG. Reproduced with permission [79]. Copyright 2022, Wiley-VCH.

With the advancement of PSCs commercialization and the increasing emphasis on environmental protection and experimental safety issues, the toxicity of a series of polar aprotic solvents (DMF, DMSO, NMP, etc.), which are most widely used as perovskite precursor solvents, has been gradually discussed. More and more green, non-toxic solvents with controlled lattice growth have been introduced into PSCs systems [71]. Chen et al. used tin oxide nanorods (SnO_2_-NRs) as ETL substrates and used green solvent triethyl phosphate (TEP) as the main solvent of the perovskite precursor to prepare perovskite films [72]. SnO_2_-NRs can promote the nucleation process and delay the perovskite crystallization rate by providing a large number of heterogeneous nucleation sites with reduced Gibbs free energy. The strong interaction between the green solvent TEP and PbI_2_ can slow down the crystal growth rate. A perovskite film with uniform morphology and large grain size was prepared, and the perovskite defects were effectively passivated. Gao et al. first reported the use of a green, non-toxic Lewis base solvent *N*-formylmorpholine (NFM) to replace toxic DMF [73]. The interaction between NFM and PbI_2_ is stronger than that of DMF, which is beneficial to inhibiting the rapid crystallization of PbI_2_ and delaying crystal growth. Besides, NFM has a higher viscosity than DMF, and the slow evaporation rate can lead to a wider anti-solvent drop window for crystal growth, which provides favorable conditions for the formation of dense and smooth high-quality perovskite films (Figure 5a). The trap state density of PbI_2_@NFM-based PSCs was significantly reduced, and the trap recombination and non-radiative recombination of perovskite were effectively suppressed. The final champion PCE reached 22.78%, while the PCE based on PbI_2_@DMF solvent was 21.97%. In addition, the humidity stability of PbI_2_@NFM-based PSCs is greatly enhanced, and it still maintains more than 90% of their initial efficiency after aging for more than 30 days at a relative humidity of ~35% in ambient air.

In recent years, ionic liquids (ILs) have attracted much attention in the emerging solvents of perovskite precursors. ILs are a kind of low melting point salt (*T_m_* < 100 °C). It has excellent physical and chemical properties and has good compatibility with PSCs. ILs have an excellent liquid range and thermal working range, which can reach 300 °C from −90 °C in some cases [80,81]. The treatment of perovskite precursor solutions usually requires a wide temperature range, so ILs work well in the solvent engineering of PSCs. In addition to the interactions that exist in conventional organic solvents (hydrogen bonding, Van der Waals interactions, etc.), ILs also have specific ionic interactions (electrostatic attraction or repulsion of charged particles), which allow ILs to be mixed with a wide range of polar substances and dissolve both organic and inorganic substances. The cations in ILs have alkyl chains of different lengths, which is beneficial to improve their solubility in less polar fluids [82,83]. The solubility of the solvent in the perovskite precursor has an important influence on the crystallization process and quality of the prepared film, so ILs can be used as a perovskite precursor solvent.

In the past, the preparation of high-quality perovskite films must be carried out in an inert atmosphere, and the temperature and humidity must be strictly controlled. Huang et al. first reported the use of ionic liquid methylamine acetate (MAAc) to replace the traditional solvent DMF as the perovskite precursor to prepare high-quality MAPbI_3_ films in ambient air [84]. Unlike DMF, MAAc dissolves perovskite precursors by forming Pb-O strong chelates and N-H---I hydrogen bonds with PbI_2_, so it has a stronger ability to induce directional crystallization and chemically passivate grain boundaries. The unique molecular structure of MAAc ultimately improves the quality of perovskite films and the performance and stability of devices. The PSCs based on MAAc solvent achieve a champion efficiency of 21.18%. In another work, Huang et al. synthesized stable black *α*-FAPbI_3_ in ambient air using methylamine formate (MAFa) as a precursor solvent (Figure 5b) [74]. During the formation of PbI_2_ thin films, the strong chelation between the C=O group and Pb^2+^ leads to the regular arrangement of PbI_2_ crystals, forming a series of PbI_2_ crystal structures with nanoscale “ion channels” and growing perpendicularly to the substrate. These channels accelerate the entry of FAI into the interior of PbI_2_ thin films and react with PbI_2_ to form stable black phase *α*-FAPbI_3_ perovskite thin films. Meanwhile, the formate ions remaining at the crystallization site can anchor the defects in situ, reducing the possibility of film defect formation, and the surface roughness of the perovskite film decreases from 20.5 nm to 10.1 nm. In addition, the trap state density of FAPbI_3_@MAFa perovskite film is also significantly lower than that of FAPbI_3_@DMF/DMSO perovskite film, which is more favorable to reduce the probability of carrier recombination (Figure 5c). Finally, the PSCs device based on MAFa solvent has 24.1% PCE, which is much higher than 22.1% based on DMF/DMSO solvent.

### 3.2. Precursor Additive Engineering

In addition to precursor solvent engineering, additive engineering that can increase grain size, passivate defects, and improve carrier extraction and transport to suppress SRH non-radiative recombination is also an important passivation strategy. The additives in the perovskite precursor solution can regulate the crystallization of perovskite, stabilize the phase state of perovskite, passivate the defects of perovskite, and optimize the interface morphology as well as the energy level of perovskite [85,86,87]. At present, there are many kinds of additives used in PSCs, such as ILs, polymers, and small organic molecules. The diversity of available additives is mainly due to the good coordination ability of anions and cations in halide perovskites, which is the basis for the solution processing of halide PSCs.

Taima et al. first used ILs as the precursor additive of MAPbI_3_ perovskite by adding 1 wt% 1-hexyl-3-methylimidazolium chloride (HMImCl) [88]. Compared with the original MAPbI_3_ film, the film after ILs treatment is smooth and uniform, which provides a new idea for the production of high-quality perovskite films. Inspired by this work, Snaith et al. presented an inverted mixed cation PSCs with 1-butyl-3-methylimidazolium tetrafluoroborate (BMIMBF_4_) as an additive [75]. They were surprised to find that in the BMIMBF_4_-modified perovskite film, BF_4_ is mainly located in the embedded interface, while BMIM exists in the whole bulk film and accumulates in the embedded interface (Figure 5d,e). This result indicates that [BMIM]^+^ and [BF_4_]^−^ ions accumulate at the perovskite/NiO*_x_* interface. The improvement of PSCs performance is mainly due to the presence of BMIM, and BF_4_ ensures that the introduction of ILs does not negatively affect film performance and device performance. The final champion PCE reached 19.80%, and the environmental stability of the device was greatly improved. The PCE was only reduced by 14% after 100 h of aging under full-spectrum sunlight at 60–65 °C.

The protonated amine carboxylic acid ILs mentioned in the previous section have also received extensive attention in additive engineering due to their unique molecular structure and high solubility. Zhang et al. innovatively reported the crystallization kinetics control of MAPbI_3_ perovskite precursor additive MAAc in carbon-based mesoporous PSCs [89]. The crystallinity of MAPbI_3_ film modified by MAAc increases obviously, and the defect density decreases. In addition, they further elucidated the effect of MAAc on crystal growth kinetics by Fourier transform infrared (FTIR). The results show that MAAc has an effective coordination effect on non-coordinated Pb^2+^ defects, which is beneficial to inhibit the non-radiative recombination of carriers and promote charge transfer in the device. Similar to MAAc, butylammonium acetate (BAAc) is also a protonated amine carboxylic acid ILs containing acetate anions (Ac^−^). Recently, Yang et al. added BAAc as an additive in the PbI_2_@DMF solution to adjust perovskite crystallization by strong bonding interaction with the PbI_2_ precursor solution and obtained high-quality perovskite films with significantly increased grain size [76]. The results of GIWAXS verify that the BAAc perovskite film has a high diffraction intensity along the (110) ring at *q* = 10 nm^−1^ (Figure 5f,g). The diffraction rings at 11 nm^−1^ and 16 nm^−1^ in the perovskite film doped with BAAc are significantly suppressed. These phenomena indicate that the chemical bond between BAAc and [PbI_6_]^4−^ skeleton forms directional crystallization, which further indicates that BAAc has the effect of regulating the crystallization kinetics of perovskite. In addition, they found that the defects of perovskite films prepared by doping BAAc were significantly reduced and the performance and stability of the devices were improved. Finally, the best device prepared by BAAc has a PCE of 20.1%, V_OC_ of 1.12 V, FF of 79%, and J_SC_ of 22.7 mA cm^−2^.

Polymers have become one of the most effective passivation additives for PSCs due to their special functional groups. Some atoms (S and N) in the polymer can react with Pb^2+^ in the perovskite to stabilize the perovskite structure and improve the crystallinity and morphology of the perovskite film. In addition, due to its excellent hydrophobicity, thermoplasticity, electrical conductivity, and mechanical stability, the addition of polymers can effectively reduce the sensitivity of perovskite materials to water, oxygen, temperature, and ultraviolet radiation, which helps to improve the stability of the device [90]. Therefore, polymers as indispensable PSCs additives have been extensively explored in regulating the nucleation and crystallization processes of perovskite films and improving device performance.

Su et al. first used PEG as a perovskite precursor additive to prepare high-quality perovskite films [91]. They found that the morphology of PEG-modified films was greatly improved, the surface was smoother, no obvious holes and the roughness was significantly reduced. This is mainly because PEG can slow down the growth and aggregation of perovskite crystals during nucleation and reduce the gap between perovskite grain boundaries during phase transformation. The optimized perovskite film has higher absorption to promote charge transfer, which greatly improves V_OC_ and J_SC_. Cheng et al. introduced polyvinyl alcohol (PVA), poly (methyl acrylate) (PMA), and polyacrylic acid (PAA) as additives into MAPbI_3_ PSCs to explain the role of different functional groups (-OH in PVA, -C=O in PMA and -COOH in PAA) in the passivation process of additives [92]. The FTIR spectra of MAPbI_3_ films doped with three different additives showed that the -OH peak of PVA-MAPbI_3_ and the -C=H peak of PMA-MAPbI_3_ had a red shift. They explained that -OH in PVA and MA^+^ in MAPbI_3_ formed hydrogen bonds, and -C=O in PMA complexed with uncoordinated Pb^2+^ in MAPbI_3_. In addition, the shift of -OH and -C=H in PAA-MAPbI_3_ is more obvious than that in PVA-MAPbI_3_ and PMA-MAPbI_3_. They believe that the -COOH of PVA can not only selectively interact with MA^+^ and I^−^ through hydrogen bonds but also complex with uncoordinated Pb^2+^ to more effectively passivate defects. Finally, PAA-modified MAPbI_3_ PSCs achieved a champion PCE of 20.29% and a V_OC_ of 1.13 V in all modified devices.

Most polymers contain one or two passivation functional groups. Due to the complex synthesis process and harsh experimental conditions, polymers with three or more passivation functional groups are rarely reported. Polyamide derivatives (PAB) is a rare polymer containing three functional groups (hydroxyl, secondary amine, and carboxyl), which is synthesized by a novel multicomponent reaction between benzoxazine-isocyanide chemistry (BIC). Ling et al. first used phenolic hydroxyl substituted PAB as precursor solution additives to passivate the perovskite active layer [77]. They found that the hydroxyl and carboxyl groups in PAB can act as Lewis bases to react strongly with Pb^2+^ in perovskite, thereby passivating defects. At the same time, the N atom in the secondary amine can coordinate with I^−^ due to its power supply characteristics. The interaction of these functional groups with the perovskite material effectively suppresses the non-radiative recombination of the carriers (Figure 5h), ultimately increasing the champion efficiency of PSCs from 19.45% to 21.13%.

Unlike large-sized polymers, small organic molecules have attracted much attention in PSCs additive engineering due to their small size and ability to enter perovskite lattices for passivation. Fullerene was first discovered by Smalley et al. in 1985. Because of its unique physical and chemical properties, it has attracted wide attention from the scientific community, including the photovoltaics industry [93]. Fullerenes and its derivatives are very suitable for PSCs due to their unique high electron mobility and surprisingly small recombination energy [94]. Nowadays, fullerene-based materials have been widely used as electron transfer layer materials and interface defect passivation materials for PSCs [95,96]. The role of fullerenes and their derivatives in PSCs additive engineering is also crucial. After entering the perovskite lattice, these small molecules can completely cover the surface of the perovskite grain boundary and act on ions attempting to move along the grain boundary by physical blocking [97]. Wu et al. systematically compared and analyzed a series of fullerene derivative additives, such as C_60_, PCBM, and C_60_-taurine (C_60_-Ta), and added them to perovskite precursors to construct perovskite-fullerene heterojunction PSCs [78]. Compared with PCBM, the energy levels of C_60_ and C_60_-Ta are more matched with the energy levels of perovskite (Figure 5i), thereby enhancing the electron transfer of perovskite, inhibiting the carrier recombination and prolonging the carrier lifetime of perovskite. Finally, compared with the control device with a PCE of 14.87%, the efficiency of all fullerene modification devices was improved. The PCE of PSCs with C_60_-Ta was 16.46%, which was slightly lower than 16.59% of C_60_ PSCs but higher than 15.94% of PCBM-based PSCs. In addition, they also studied the effect of chemical composition in fullerene derivatives on the performance of PSCs device parameters, trying to explain the specific role of the C_60_ cage and grafted side chain in fullerene and its derivative additives. C_60_ has higher carrier mobility than C_60_-Ta, but the grafted side chain of C_60_-Ta can more effectively improve crystal quality and reduce defects, thereby further improving device stability. It is worth noting that the grafting side chain of C_60_-Ta has a negative effect on carrier migration, resulting in a lower final efficiency of C_60_-Ta-modified devices than that of C_60_-modified devices. Therefore, it is very important to select a suitable C_60_ grafted side chain to simultaneously reduce the perovskite defect state and improve carrier transport to balance the stability and PCE of PSCs. Jeon et al. innovatively proposed a new method to introduce [6,6]-phenyl-C_61_-butyric acid 2-[2-(2-methoxyethoxy)ethoxy]ethyl ester (PC_61_B-TEG) into perovskite devices and induce favorable vertical gradients [79]. Because the TEG on fullerene can significantly improve its solubility in the polar solvent of perovskite precursor, the charge transfer ability and grain defect passivation ability of the modified perovskite film is significantly enhanced. FTIR results show that when the fullerene derivative is mixed with PbI_2_ (Figure 5j), the peaks of C=O and C-O in PC_61_B-TEG move downward, indicating that the additive effectively passivates Pb^2+^, indicating that the vertical gradient PC_61_B-TEG additive can effectively passivate the defect sites of perovskite, and the coated PC_61_B-TEG interface also significantly enhances the carrier transport capacity. Finally, it is found that devices based on different perovskites exhibit higher performance parameters than conventional devices. For MAPbI_3_-based devices, the PCE of devices with PC_61_B-TEG added increased from 17% to 19.5%. The device based on FA_0.65_ MA_0.35_ PbI_3-*x*_Cl*_x_* has more significant performance improvement. Compared with the traditional device with a V_OC_ of 1.14 V, J_SC_ of 24.97 mA cm^−2^, FF of 79%, and PCE of 21.88%, the improved device has a V_OC_ of 1.13 V, J_SC_ of 25.42 mA cm^−2^, FF of 81% and PCE of 23.34%, which is also the highest certification efficiency for PSCs prepared by fullerene derivative additives so far.

### 3.3. Anti-Solvent Engineering

**Figure 6:**
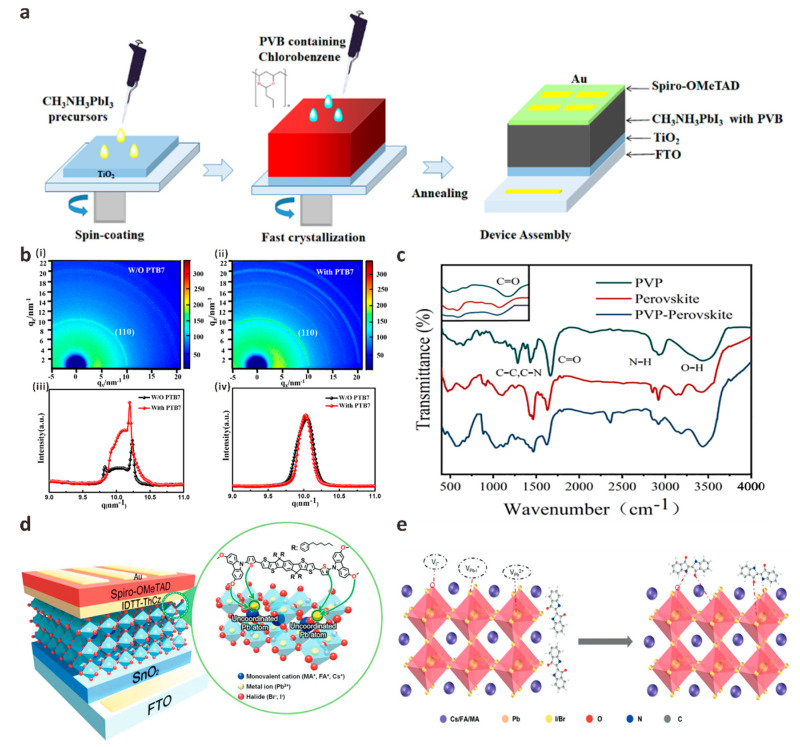
(**a**) One-step preparation of PSCs schematic, in which PVB is introduced into the perovskite layer by anti-solvent engineering. Reproduced with permission [99]. Copyright 2021, Elsevier. (**b**) 2D-GIXRD diagram of (**i**) original and (**ii**) PTB7 modified perovskite films at a grazing incidence angle of 0.06° and azimuthal integral intensity diagram of (**iii**) perovskite (110) plane and (**iv**) perovskite (110) diffraction peak. Reproduced with permission [100]. Copyright 2022, Elsevier. (**c**) FTIR spectra of PVP, perovskite and PVP-modified perovskite films. Reproduced with permission [101]. Copyright 2022, Elsevier. (**d**) The interaction diagram of IDTT-ThCz molecule with perovskite layer. Reproduced with permission [102]. Copyright 2021, Wiley-VCH. (**e**) Indigo and mixed hybrid perovskite passivation mechanism diagram. Reproduced with permission [103]. Copyright 2022, Wiley-VCH.

Anti-solvent engineering is another way to introduce additives into the perovskite light-absorbing layer for component regulation. Anti-solvents, such as chlorobenzene (CB), are a type of non-polar solvents that are miscible with the deposition solvent in the perovskite precursor solution and insoluble with perovskite salts. It plays an important role in the surface morphology and crystallization properties of perovskite films. After the perovskite precursor solution is spin-coated on the substrate for a specific time, an anti-solvent is added dropwise to the rotating precursor solution to prepare a perovskite film. The film produced by this method is smoother and of higher quality than the film prepared by spin-coating without adding a solvent dropwise [98]. Anti-solvent engineering provides a practical way to passivate the carrier non-radiative recombination problem and suppress its defects as much as possible. But dropping anti-solvent on the perovskite layer results in a fast and uncontrollable crystallization process and produces a large number of grain boundaries and surface defects. Wang et al. added polyvinyl butyral (PVB) as an additive to the anti-solvent CB when preparing MAPbI_3_ films by one-step spin-coating process, which was added to the film surface before the end of spin-coating to help the perovskite crystal growth to improve film quality (Figure 6a) [99]. They found that the grain size of the modified perovskite film increased significantly to 600–700 nm, and the number of grain boundaries decreased significantly, which was beneficial to the reduction of the defect density of the film. Gao et al. added poly{4,8-bis[(2-ethylhexyl)oxy] benzo [1,2-b:4,5-b’]dithiophene-2,6-diyl-alt-3-fluoro-2-[(2-ethylhexyl)carbonyl]thieno[3,4-b]thiophene-4,6-diyl} (PTB7) as an additive to the anti-solvent CB of perovskite film to fully study the passivation mechanism of PTB7 [100]. They found that the introduction of PTB7 significantly reduced defects in perovskite films and increased crystallinity. The synchrotron radiation grazing incidence X-ray diffraction (GIXRD) results of perovskite films before and after adding PTB7 at different grazing incidence angles and different detection depths also confirm this conclusion (Figure 6b). Gao et al. explained that this phenomenon is due to the large size of the perovskite surface that does not enter the perovskite lattice and only exists at the grain boundary or on the surface of the film. The PTB7 molecule interacts with the Pb atom in the precursor to form a Lewis base coordination bond, thereby slowing down the crystallization kinetics of the film nucleation and increasing the crystallinity. Based on the starting point of green environmental protection, Wang et al. added non-toxic polymer polyvinylpyrrolidone (PVP) as an additive to the green anti-solvent isopropanol to passivate perovskite films [101]. Time-resolved photoluminescence (TRPL) spectra show that the non-radiative linear recombination of carriers in the passivated device is significantly reduced, mainly due to the lone pair electrons in PVP can share C=O bonds with uncoordinated Pb^2+^ in perovskite to inhibit ion migration and stabilize perovskite crystal structure (Figure 6c).

In addition to polymers, small organic molecules are also used in anti-solvent engineering. Song et al. first synthesized an indacenodithieno[3,2-b]thiophene-based small molecule (IDTT-ThCz) and introduced it as an additive anti-solvent into perovskite to assist crystallization (Figure 6d) [102]. Finally, PSCs with high PCE and obvious thermal stability were prepared. The FF of the modified device is as high as 80.4%, and the PCE is as high as 22.5%. Simultaneously, 95% of the initial PCE can be retained after 500 h storage under thermal conditions (85 °C). As they explained, the Lewis atoms in IDTT-ThCz can react with Pb^2+^ in the perovskite precursor, passivate the electronic defect state and effectively inhibit the degradation of the perovskite layer. Due to the unique p-type semiconductor characteristics of IDTT-ThCz, the charge extraction capability of PSCs has also been significantly improved. Ma et al. first reported a natural small organic dye molecule Indigo as a passivator for the design and preparation of high-quality hybrid perovskite films through anti-solvent engineering [103]. They treated the Cs_0.05_FA_0.85_MA_0.10_Pb(I_0.90_Br_0.10_)_3_ perovskite film by dissolving the Lewis base indigo molecule in CB at an optimal concentration and proved that the presence of the C=O/-NH functional group has a significant effect on the passivation of the original perovskite film defects. The carbonyl group (electron-pair donor) in the indigo molecule can interact with the uncoordinated Lewis acid Pb^2+^ on the perovskite surface and the Pb-I antisite defect, and the amino group can interact with the I-site. In addition, the hydrogen bond between indigo molecules and the perovskite surface can inhibit ion migration and further passivate perovskite film defects (Figure 6e). Therefore, the champion PCE of PSCs passivated by indigo increased from 20.18% to 23.22%.

## 4. Dopant Passivation

Although introducing additives directly into a perovskite precursor solution can reduce the trap-state density of the film and suppress non-radiative recombination of carriers, this passivation strategy carries the risk of introducing impurities into the perovskite crystal that affects the long-range ordered structure of the perovskite crystal [104]. Therefore, the researchers turned the passivation target to the carrier transport layer adjacent to the perovskite layer. The elemental doping dopant passivation project on the carrier transport layer can promote the carrier transport rate and adjust the energy level barrier between the interfaces, which will further passivate the perovskite film defects, control the crystallization process of the perovskite film and increase the crystallinity.

### 4.1. ETL Doping

**Figure 7:**
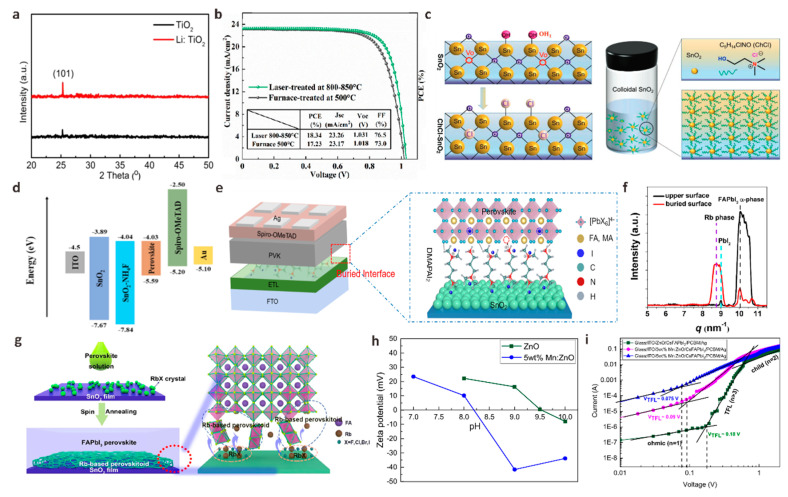
(**a**) X-ray diffraction (XRD) patterns of TiO_2_ and Li: TiO_2_ samples. Reproduced with permission [105]. Copyright 2023, Elsevier. (**b**) J-V curves of Cs_0.1_FA_0.9_PbI_3_ devices based on furnace-treated and optimized laser-processed Ta-TiO_2_ films. Reproduced with permission [106]. Copyright 2022, American Chemical Society. (**c**) The surface defect sites of SnO_2_ and the schematic diagram of ChCl-SnO_2_ nanoparticle solution. Reproduced with permission [107]. Copyright 2022, Wiley-VCH. (**d**) Energy level diagram of PSCs before and after NH_4_F modification. Reproduced with permission [108]. Copyright 2022, American Chemical Society. (**e**) PSCs device structure diagram and DMAPAI_2_ embedded interface diagram. Reproduced with permission [109]. Copyright 2022, Elsevier. (**f**) Basic integral intensity corresponding to GIWAXS data of RbCl modified film upper surface and buried surface. (**g**) A schematic diagram of RbX crystallized on the surface of SnO_2_ and Rb-based perovskite formed on the buried interface of perovskite film. Reproduced with permission [110]. Copyright 2022, American Chemical Society. (**h**) IEP measurements of ZnO and Mn:ZnO powders. (**i**) SCLC measurements for ZnO and Mn:ZnO-based electron-only devices. Reproduced with permission [111]. Copyright 2022, American Chemical Society.

The biggest disadvantage of TiO_2_ is the low electron mobility, which greatly limits the performance parameters of the device. By doping alkali metals or transition metals to passivate the dense TiO_2_ layer and/or mesoporous TiO_2_ layer, the electronic band structure and the trap state of TiO_2_ will be changed. It is very helpful to improve the charge transport performance of the device. Inspired by previous studies, Chu et al. used metal Li ions doped into TiO_2_ (Li-TiO_2_) as a new ETL for carbon-based CsPbIBr_2_ PSCs [105]. They found that the optical band gap of TiO_2_ did not change after Li doping, but the crystallinity of TiO_2_ films could be effectively improved (Figure 7a). At the same time, the carrier recombination at the Li-TiO_2_/CsPbIBr_2_ interface is suppressed, which greatly improves the efficiency and stability of inorganic CsPbIBr_2_ PSCs. Liu et al. developed a fast one-step laser-assisted doping process to incorporate the transition metal tantalum (Ta) into the TiO_2_ ETL (Ta-TiO_2_), inducing the crystallization of the TiO_2_ film from its amorphous precursor to the anatase phase [106]. The conductivity and electron transport capacity of the TiO_2_ film treated by the best laser process is improved, and the high concentration of Ti^3+^ defects on the surface of the film is effectively suppressed. The perovskite film with Ta-TiO_2_ ETL as the substrate has good coverage and crystallinity while reducing the non-radiative recombination of carriers. The MA_0.1_FA_0.9_PbI_3_ PSCs device based on Ta-TiO_2_ ETL finally achieved a champion PCE of 18.34%, mainly due to a significant increase in FF from 73% to 76.5% (Figure 7b).

As an emerging ETL, SnO_2_ has attracted much attention due to its low-temperature solution treatment and high electron mobility (100–200 cm^2^ V^−1^ s^−1^). However, because of the absence of high-temperature sintering, there are a large number of oxygen vacancies on the SnO_2_ film. SnO_2_ nanoparticles easily form agglomerates in the solution state, which often leads to a large number of intrinsic defects, resulting in poor film uniformity, crystallinity, and then obvious leakage current. Meanwhile, the uncoordinated Sn will hang on the surface of SnO_2_ and become a trap state of electrons in the conduction band, forming a potential barrier to hinder electron transport [112]. It is necessary to use dopants with different characteristics to modify SnO_2_ to passivate surface defects so that ETL can form effective contact with perovskite and reduce interface carrier recombination. Ammonium salt is a commonly used SnO_2_ dopant. Zhang et al. developed a molecular bridge strategy to change the properties of the buried interface in n-i-p PSCs by introducing a multifunctional dopant 2-Hydroxyethyl trimethylammonium chloride (ChCl) into SnO_2_ ETL (Figure 7c) [107]. The multifunctional molecular structure (NH_4_^+^, Cl^−^, -OH) in the dopant ChCl can be used as a molecular bridge to passivate defects in colloidal SnO_2_ and simultaneously regulate perovskite crystallization. Therefore, the perovskite film has larger grains, high uniformity and low defects, which is beneficial for suppressing non-radiative recombination and reducing voltage loss. At the same time, the embedded ChCl-SnO_2_ ETL also exhibits reduced defect state density, matched energy levels and high conductivity. Therefore, at a significant V_OC_ of up to 1.193 V, the device PCE increased significantly from 20.0% to 23.07%. Similar to this work, Liu et al. proposed a modification of ETL by incorporating organic ammonium salt propylammonium chloride (PACl) into SnO_2_ colloidal solution to study the interaction mechanism between organic salts and alkaline colloidal solution [113]. PACl can passivate perovskite layer defects and enhance the crystallization of perovskite films. The main reason is that Cl^−^ and PA^+^ will be introduced after PACl is incorporated into SnO_2_ colloidal alkaline solution, and Cl^−^ will diffuse to the PbI_2_ layer to promote perovskite nucleation and increase perovskite grain size. PA^+^ can passivate grain boundaries and reduce perovskite film defects. Therefore, the overall performance of the device based on SnO_2_-PACl ETL has been significantly improved. Champion device PCE was 22.27%. The incorporation of PACl also significantly improved the stability of PSCs, and the PCE remained 85% of the original value after 800 h in air. Chang et al. introduced a method to effectively passivate the surface defects of SnO_2_ thin films by doping ammonium fluoride (NH_4_F) into SnO_2_ precursor [108]. The F in NH_4_F can repair the terminal hydroxyl defects on the surface of SnO_2_ and reduce the defects on the surface of SnO_2_ and perovskite. The terminal hydroxyl groups on the surface of SnO_2_ have been confirmed to act as defect sites to introduce deep level defects into the band gap, thus causing carrier recombination between interfaces. The doping of NH_4_F makes the energy level configuration of the device more conducive to electron extraction (Figure 7d). The results show that the PSCs based on SnO_2_-NH_4_F ETL reaches 22.12% PCE, and the V_OC_ is 70 mV higher than that of the control device.

In addition to chloride ammonium salt and fluoride ammonium salt, iodide ammonium salt is also a kind of commonly used organic ammonium salt. Shi et al. reported a strategy to passivate the SnO_2_ layer using an asymmetric diammonium salt N, N-dimethyl-1,3-propanediamine dihydroiodide (DMAPAI_2_) (Figure 7e) [109]. The I^−^ in DMAPAI_2_ can passivate V_O_ on the surface of SnO_2_ by electrostatic coupling, thereby enhancing the electron mobility of SnO_2_ and adjusting the energy level structure. The ammonium cations on the surface of DMAPAI_2_-SnO_2_ can interact with iodides in perovskite precursors through ionic bonds and/or hydrogen bonds to slow down the growth process of perovskite, which is conducive to promoting uniform nucleation and growth. Based on this strategy, the PCE of PSCs increased significantly from 20.78% to 23.20%.

Inorganic halide salts are also effective ETL dopants for preparing efficient PSCs. Wu et al. incorporated rubidium chloride (RbCl) into the SnO_2_ precursor solution and crystallized an island pattern on the surface of SnO_2_ based on the “rigid skeleton” structural properties of Rb [110]. In the process of perovskite crystal growth, RbCl crystal can be used as a nucleation center to act as a “scaffold” to anchor the uncoordinated atoms on the surface of the perovskite film to reduce defects (Figure 7f,g). They found that the grain size and crystallization strength of SnO_2_-RbCl-based perovskite films were greatly improved. The simulation results also confirmed that the perovskite termination layer based on SnO_2_-RbCl could significantly inhibit the formation of surface iodide vacancies, which contributes to the crystallization and passivation of perovskite films. The enhancement of defects leads to a slower carrier recombination rate in the perovskite film, which further reduces the non-radiative recombination of the perovskite surface and improves device stability. Finally, PSCs prepared by the RbCl dopant showed 25.14% champion PCE.

ZnO with high electron mobility (120 cm^2^ V^−1^ s^−1^) is also an excellent ETL material for planar p-i-n structure PSCs, which has higher light transmittance and better conduction band offset similar to TiO_2_ and SnO_2_ [114]. However, the high alkalinity of ZnO and the higher isoelectric point (IEP) than other metal oxides make MA^+^ and FA^+^ in the perovskite film rapidly deprotonated, thereby increasing more defect sites and result in higher charge recombination at the interface. In this concern, Krishnamoorthy et al. first doped solution-treated Mn into ZnO to adjust its IEP [111]. They found that the IEP (~8.2) of Mn: ZnO was significantly lower than that of the original ZnO (~9.5). X-ray photoelectron spectroscopy (XPS) analysis also shows that the formation of OH peaks and oxygen vacancies in modified ZnO is relatively low. SCLC measurement shows that the trap state density of PSCs based on Mn: ZnO is significantly reduced, which indicates that the doping of Mn reduces the defects of ZnO and perovskite films, thus ensuring better electron transport between perovskite and ZnO (Figure 7h,i). Finally, the PCE of Mn: ZnO-based PSCs increased from 11.7% to 13.6%, which was about 15% higher than that of the original ZnO-based PSCs. Akram et al. doped Al into ZnO to improve carrier mobility to suppress the generation of deep-level defects [115]. When 1% or 2% Al is doped in ZnO, the lattice shrinkage leads to an increase in grain size, the uniformity, and the smoothness of the film. The surface defects are effectively passivated. Because Al^3+^ replaces Zn^2+^ in the lattice site, the carrier concentration increases, indicating that the ETL/perovskite interface can achieve more effective charge extraction.

### 4.2. HTL Doping

**Figure 8:**
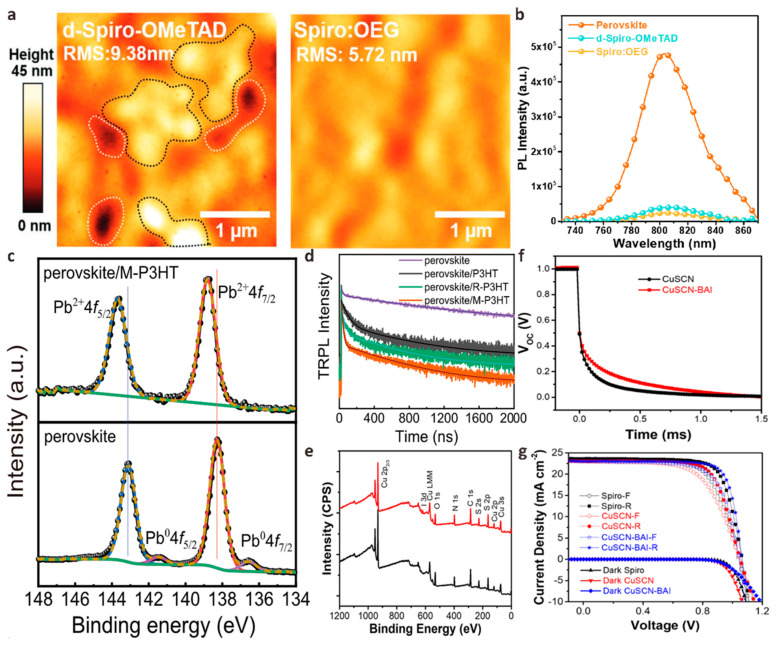
(**a**) AFM images of Spiro-OMeTAD and Spiro:OEG films coated on perovskite, where black dashed lines indicate aggregation of Li-TFSI and white dashed lines indicate pinholes. (**b**) PL spectra of perovskite, perovskite and Spiro-OMeTAD or Spiro:OEG HTL. Reproduced with permission [121]. Copyright 2022, Wiley-VCH. (**c**) High-resolution XPS spectra of Pb 4f spectra and (**d**) TRPL spectra of M-P3HT modified perovskite films. Reproduced with permission [122]. Copyright 2022, Springer Nature. (**e**) XPS full measurement spectra of CuSCN and CuSCN-BAI films. (**f**) Transient photovoltage decay (TPV) curves and (**g**) dark and light J−V curves of PSCs based on CuSCN and CuSCN-BAI films. Reproduced with permission [123]. Copyright 2022, American Chemical Society.

Spiro-OMeTAD doped with lithium bis(trifluoromethanesulfonyl)imide (Li-TFSI) and 4-*tert*-butylpyrimidine (tBP) is considered to be the most effective HTL material for planar n-i-p structure PSCs with many record PCE [116,117,118,119]. However, Li-TFSI/tBP doped spiro-OMeTAD has poor environmental conductivity, which cannot effectively passivate the perovskite/HTL interface and reduce perovskite crystal defects. The unstable HTL compositions and iodide salts will cause serious degradation of the device, resulting in unstable device performance. Overcoming these shortcomings of the spiro-OMeTAD layer can effectively improve the performance and stability of PSCs. Chen et al. designed a passivation strategy to incorporate multi-walled carbon nanotubes (MWCNT:NiO*_x_*) modified with multifunctional NiO*_x_* quantum dots (QD) into spiro-OMeTAD [120]. Due to the strong interaction between O in MWCNT:NiO*_x_* and H in spiro-OMeTAD, the conductivity of modified HTL is improved. The anchoring effect of the Li-O bond in MWCNT:NiO*_x_* on Li-TFSI effectively limits the migration of Li^+^ ions. MWCNT:NiO*_x_* also passivates perovskite crystal defects by forming Ni-I bonds with perovskite. The interface defects can be reduced, and the extraction and transfer of holes are promoted. The PSCs device fabricated by this passivation strategy has a PCE of up to 22.73%, which is 1.2 times greater than that of the original spiro-OMeTAD-based PSCs. The environmental, thermal, and light stability are improved significantly. To further enhance the stability of spiro-OMeTAD, Li et al. designed N2, N2′, N7, N7′-tetrakis (4-((2-methoxyethoxy) methyl) phenyl) -tetra(yridine-4-yl)-9,9′-spirobi[fluorene]-2,2′,7,7′-tetraamine (spiro-BD-2OEG) composed of a main chain of spirobifluorene (spiro), a terminal group of phenylpyridine-4-amine (BD) and oligo (ethyl-eneglycol) (OEG) side chain [121]. They found that spiro-BD-2OEG provides a strong π-π interaction between the easily reduced benzene ring and the pyridine group of tBP, thereby further inhibiting the volatilization of tBP compared with spiro-OMeTAD. The lone pair electrons of the pyridine part of spiro-BD-2OEG combine with Li^+^ to accelerate its dissolution, which is beneficial in inhibiting morphological defects and stabilizing the composition. Spiro-BD-2OEG doped spiro-OMeTAD film has long-range ordered molecular order and low roughness, which helps to form strong electronic contact with perovskite film and improve stability. The photoluminescence (PL) mapping strength of the perovskite modified by spiro-BD-2OEG was significantly reduced, indicating that the surface defects of the perovskite were effectively passivated and the non-radiative recombination at the interface was inhibited (Figure 8a,b). Finally, the PSCs device based on piro-BD-2OEG doped spiro-OMeTAD achieved an excellent PCE of 24.19%.

Compared with the classic spiro-OMeTAD, poly (3-hexylthiophene) (P3HT) is a lower-cost and more stable HTL material in planar n-i-p structure PSCs. However, the alkyl side chain of P3HT will directly contact the perovskite film, resulting in poor electron contact at the P3HT/perovskite interface, which exacerbates the non-radiative recombination of PSCs and makes the PCE of pure P3HT-based PSCs generally low [124]. To solve this problem, Gao et al. used 2-((7-(4-(bis(4-methoxyphenyl)amino)phenyl)−10-(2-(2-ethoxyethoxy)ethyl)−10*H*-phenoxazin-3-yl)methylene)-malononitrile (MDN) to modify P3HT to improve the bad contact between perovskite and P3HT [122]. The N atom in the malononitrile group in the MDN can be electrostatically coupled with the uncoordinated Pb on the perovskite surface, effectively suppressing the generation of Pb0 defects. In addition, the triphenylamine group in the MDN can form a π-π stacking with P3HT to establish a charge transport path between the perovskite/HTL. They found that the PL intensity of P3HT-modified perovskite decreased, indicating that the density of trap states at the perovskite/HTL interface decreased, and the non-radiative recombination was alleviated (Figure 8c,d). Finally, 22.87% PCE was achieved using MDN-doped P3HT as HTM, which was much higher than 12.48% of the control device.

The doping passivation engineering of HTL in planar p-i-n structure PSCs has also attracted much attention in recent years. NiO*_x_* is considered to be one of the most promising HTMs for p-i-n inverted PSCs due to its high stability, high mobility, and low cost. In addition, it has a wide band gap with high transmittance and proper VB alignment. However, compared with organic HTL materials, the biggest problem faced by NiO*_x_*-based PSCs is the lower V_OC_. In this regard, Park et al. reported the device parameters of PSCs based on modified NiO*_x_* by incorporating ammonium salt into NiO*_x_* precursor solution to study the inherent properties of modified NiO*_x_* [125]. They found that the morphology of the NiO*_x_* film doped with ammonium salt was improved, the defects were reduced, and the crystallinity was higher. The energy level and hole conductivity of NiO*_x_* are optimized, which is beneficial to hole transport. In addition, the strong interaction between ammonium salt and perovskite also optimizes the quality of perovskite films, enhances the interfacial properties between NiO*_x_* and perovskite layers, and reduces trap-assisted recombination. The MAPbI_3_ PSCs based on this new NiO*_x_* obtained a 19.91% champion PCE and an extremely high V_OC_ of 1.13 V. In addition to NiO*_x_*, CuSCN is also one of the low-cost and highly stable HTL materials in p-i-n inverted PSCs, which have attracted much attention recently [126]. The conductivity of the solution-treated CuSCN HTL is low, which is not conducive to the extraction and transmission of holes in PSCs devices. To improve the conductivity of CuSCN, Ye et al. doped *n*-butylammonium iodide (BAI) into the CuSCN precursor to optimize its p-conductivity [123]. BAI can effectively complex with Cu^2+^ in CuSCN to achieve complete coverage of the perovskite active layer. In addition, the complexation helps to generate more Cu vacancies in the CuSCN HTL, resulting in a significant increase in hole concentration and p conductivity of the CuSCN film. At the same time, the high hole extraction rate of the modified CuSCN inhibits the non-radiative recombination at the HTL/perovskite interface and achieves high device stability. Finally, the modified PSCs achieved 19.24% PCE, showing better stability than the control device in the air environment (Figure 8e–g).

## 5. Summary and Prospect

Among the third-generation solar cells, PSCs have attracted much attention due to their simple manufacturing process, low cost, and fast development. The surface defect degree of the perovskite film is one of the main factors affecting the PCE of the device, so we can improve efficiency by passivating defects. Here, we provide an in-depth review of the passivation strategies reported so far and list the detailed photovoltaic parameters of the highly efficient PSCs achieved over the past year using different passivation strategies in Table 1. Although single-junction PSCs have achieved a high PCE of 25.8%, there is still large room for further improvement from the S-Q theoretical limit of 1.6 eV band gap (30.5%). The J_SC_ of most reported high-efficiency PSCs is close to the theoretical value, while V_OC_ and FF are still lower than the theoretical value, so strategies to improve V_OC_ and FF should be reasonably formulated to further enhance PCE.

We convince that the passivation of deep defect-induced traps plays an irreplaceable role in the latest progress of PSCs device performance and stability. However, the complete understanding of the passivation mechanism has not been fully resolved, which may be largely due to the versatility of some passivators, the overall complexity of the discussed system, and limited experimental techniques. It is difficult to achieve efficient passivation in perovskite film manufacturing processes if various types of defects and their concentrations and trap depths cannot be accurately identified. This may be the biggest challenge limiting defect passivation of PSCs. Looking ahead, a better understanding of the passivation mechanism is needed to guide the selection, design, and combination of passivators to produce synergistic passivation, which is crucial for further improving the efficiency and stability of PSCs.

In addition, although lead-based PSCs show superior performance, the toxicity of lead is a common concern for all developers and consumers because lead leakage that may occur during manufacturing, installation, or disposal can seriously pollute the environment and endanger humans. Since the lattice disorder and trap density of perovskite films are proportional to the size of perovskite devices, there are still problems in the manufacture of large-area devices for commercialization. Moreover, due to the structural disorder caused by interface and grain boundary defects, PSCs are difficult to maintain good efficiency and stability in large-scale production, so much effort should be spared to developing large-scale manufacturing technologies for perovskite photovoltaics. Considering the continuous efforts of researchers and the improvement of the performance and stability of perovskite devices, the actual commercialization of PSCs seems to be fully achievable in the near future.

## Figures and Tables

**Table 1:** The passivators used in different PSC passivation strategies in the past year and the performance comparison before and after passivation.

Strategy	Passivator	Processing	*V*_OC_ [V]	*J*_SC_ [mA cm^−2^]	FF [%]	PCE [%]	*N*_trap_ [cm^−3^]	Ref.
Interface Engineering	TFPhFACl	Control	1.08	25.49	79.77	21.90	3.58 × 10^15^	[127]
		Passivated	1.16	25.42	81.26	24.00	1.44 × 10^14^	
Interface Engineering	BTACl	Control	1.08	23.78	71.74	18.43	2.74 × 10^16^	[128]
		Passivated	1.19	23.85	76.54	21.72	1.41 × 10^16^	
Interface Engineering	I-TFBA	Control	1.13	21.80	79.20	19.50	1.43 × 10^16^	[129]
		Passivated	1.18	22.80	82.20	22.02	1.14 × 10^16^	
Interface Engineering	CsF	Control	1.15	25.28	75.32	21.93	9.67 × 10^15^	[130]
		Passivated	1.18	25.47	77.31	23.13	4.83 × 10^15^	
Interface Engineering	LDA-Cl	Control	1.09	25.30	79.47	21.91	5.51 × 10^16^	[131]
		Passivated	1.13	25.46	80.69	23.28	5.09 × 10^16^	
Interface Engineering	BDDAB	Control	1.06	23.96	75.95	19.39	3.41 × 10^15^	[132]
		Passivated	1.10	24.98	80.10	22.08	1.15 × 10^15^	
Perovskite Surface Treatment	Taurine	Control	1.07	23.94	77.00	19.73	1.13 × 10^15^	[133]
		Passivated	1.13	24.72	80.20	22.54	4.70 × 10^14^	
Perovskite Surface Treatment	AHPD	Control	0.69	30.68	74.18	15.72	1.40 × 10^13^	[134]
		Passivated	0.81	30.03	79.10	19.18	7.80 × 10^12^	
Perovskite Surface Treatment	L-arginine	Control	1.13	22.98	78.56	20.37	9.98 × 10^13^	[135]
		Passivated	1.18	23.57	82.36	22.96	3.58 × 10^15^	
Perovskite Surface Treatment	FID	Control	1.08	25.31	76.80	21.07	1.29 × 10^16^	[136]
		Passivated	1.14	25.42	81.00	23.44	8.85 × 10^15^	
Perovskite Surface Treatment	ORO	Control	1.05	24.55	75.36	19.47	1.90 × 10^16^	[137]
		Passivated	1.09	24.66	76.47	20.62	1.20 × 10^16^	
Perovskite Surface Treatment	L-Theanine	Control	1.15	25.10	76.99	22.29	1.73 × 10^16^	[138]
		Passivated	1.19	25.13	81.87	24.58	0.73 × 10^16^	
Perovskite Surface Treatment	DA	Control	1.07	22.33	79.59	19.04	8.50 × 10^15^	[139]
		Passivated	1.13	23.36	83.92	22.15	3.80 × 10^15^	
Perovskite Surface Treatment	PB	Control	0.98	18.68	74.00	13.55	4.04 × 10^16^	[140]
		Passivated	1.09	21.02	79.00	18.01	2.04 × 10^16^	
Perovskite Surface Treatment	VC	Control	1.16	24.95	78.46	22.67	3.27 × 10^16^	[141]
		Passivated	1.16	25.00	79.34	23.01	1.55 × 10^16^	
Dopant Passivation	F4-TCNQ	Control	1.09	22.55	73.49	18.06	3.99 × 10^15^	[142]
		Passivated	1.11	23.70	78.58	20.67	1.16 × 10^15^	
Dopant Passivation	GV	Control	1.06	22.28	68.28	16.25	9.76 × 10^15^	[143]
		Passivated	1.09	23.33	75.73	19.20	7.17 × 10^15^	
Dopant Passivation	IMBF_4_	Control	1.09	24.66	75.07	20.18	1.96 × 10^15^	[144]
		Passivated	1.15	24.90	80.50	23.05	1.59 × 10^15^	
Dopant Passivation	LiOH	Control	1.15	22.90	73.27	19.26	3.54 × 10^15^	[145]
		Passivated	1.15	24.20	76.26	21.31	2.83 × 10^15^	
Dopant Passivation	g-C_3_N_5_	Control	1.16	22.51	73.84	19.28	2.04 × 10^16^	[146]
		Passivated	1.18	23.97	78.98	22.34	6.82 × 10^15^	

## Data Availability

Not applicable.
